# Second-generation pterocarpanquinones: synthesis and antileishmanial activity

**DOI:** 10.1186/s40409-018-0174-7

**Published:** 2018-11-29

**Authors:** Viviane dos Santos Faiões, Lívia C. R. M. da Frota, Edézio Ferreira Cunha-Junior, Julio C. F. Barcellos, Thayssa Da Silva, Chaquip Daher Netto, Silvia Amaral Gonçalves Da-Silva, Alcides J. M. da Silva, Paulo R. R. Costa, Eduardo Caio Torres-Santos

**Affiliations:** 1 Laboratório de Bioquímica de Tripanosomatídeos, Instituto Oswaldo Cruz, FIOCUZ, Av. Brasil, 4365, Pavilhao Leonidas Deane, sala 405A, Manguinhos, Rio de Janeiro, RJ 21040-900 Brazil; 2 Instituto de Pesquisa de Produtos naturais, Universidade Federal do Rio de Janeiro, Rio de Janeiro, RJ Brazil; 3 Laboratório de Imunofarmacologia Parasitária, Universidade do Estado do Rio de Janeiro, Rio de Janeiro, RJ Brazil; 4 Laboratório de Química, Universidade Federal do Rio de Janeiro, campus Professor Aloísio Teixeira, Macaé, RJ Brazil

**Keywords:** Leishmania, Pterocarpanquinone, LQ-118, Phenotypic assay, Leishmaniasis, Drug discovery, Neglected diseases

## Abstract

**Background:**

Despite the development of new therapies for leishmaniasis, among the 200 countries or territories reporting to the WHO, 87 were identified as endemic for Tegumentary Leishmaniasis and 75 as endemic for Visceral Leishmaniasis. The identification of antileishmanial drug candidates is essential to fill the drug discovery pipeline for leishmaniasis. In the hit molecule LQB-118 selected, the first generation of pterocarpanquinones was effective and safe against experimental visceral and cutaneous leishmaniasis via oral delivery. In this paper, we report the synthesis and antileishmanial activity of the second generation of pterocarpanoquinones.

**Methods:**

The second generation of pterocarpanquinones 2a-f was prepared through a palladium-catalyzed oxyarylation of dihydronaphtalen and chromens with iodolawsone, easily prepared by iodination of lawsone. The spectrum of antileishmanial activity was evaluated in promastigotes and intracellular amastigotes of *L. amazonensis*, *L. braziliensis*, and *L. infantum*. Toxicity was assessed in peritoneal macrophages and selective index calculated by CC_50_/IC_50_. Oxidative stress was measured by intracellular ROS levels and mitochondrial membrane potential in treated cells.

**Results:**

In this work, we answered two pertinent questions about the structure of the first-generation pterocarpanquinones: the configuration and positions of rings B (pyran) and C (furan) and the presence of oxygen in the B ring. When rings B and C are exchanged, we noted an improvement of the activity against promastigotes and amastigotes of *L. amazonensis* and promastigotes of *L. infantum*. As to the oxygen in ring B of the new generation, we observed that the oxygenated compound 2b is approximately twice as active against *L. braziliensis* promastigotes than its deoxy derivative 2a. Another modification that improved the activity was the addition of the methylenedioxy group. A variation in the susceptibility among species was evident in the clinically relevant form of the parasite, the intracellular amastigote. *L. amazonensis* was the species most susceptible to novel derivatives, whilst *L. infantum* was resistant to most of them. The pterocarpanoquinones (2b and 2c) that possess the oxygen atom in ring B showed induction of increased ROS production.

**Conclusions:**

The data presented indicate that the pterocarpanoquinones are promising compounds for the development of new leishmanicidal agents.

## Background

**Fig. 1:**
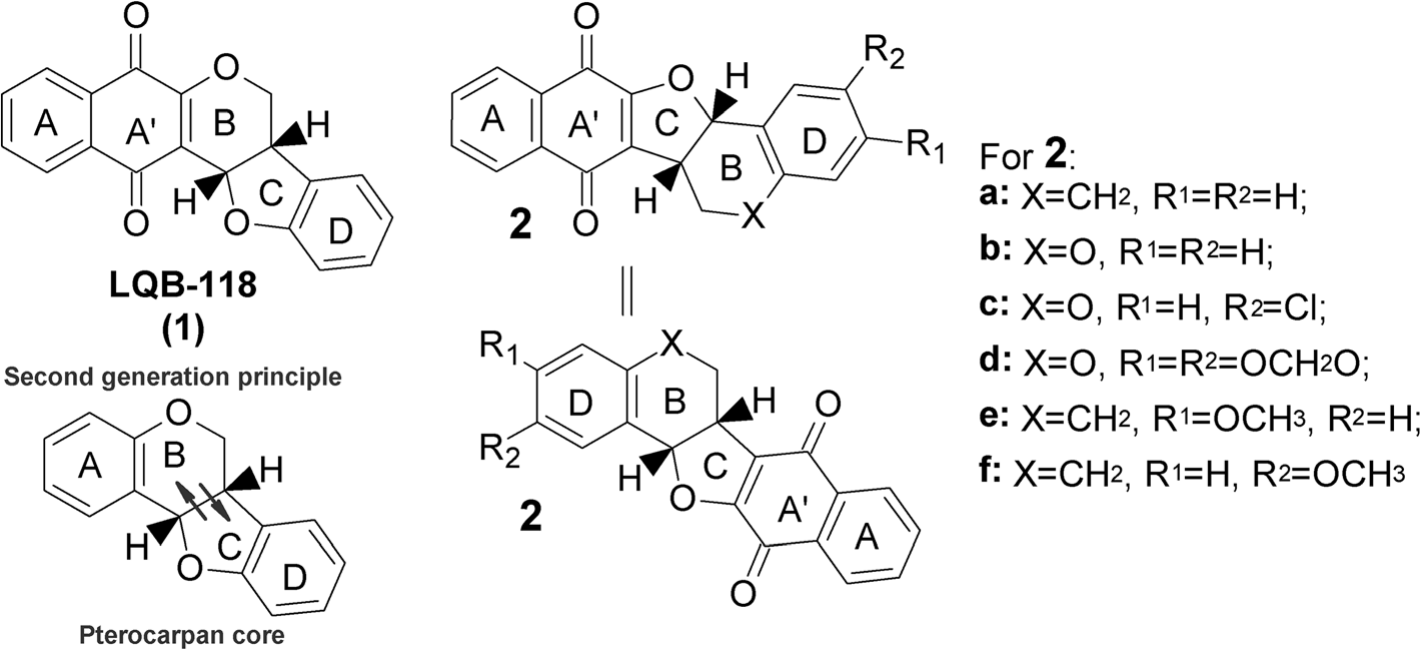
Planning of second generation pterocarpanoquinones. Design of pterocarpanquinones was based on molecular hybridization of quinone and pterocarpan core. LQB-118 (1) and second generation (2a-f)

The first generation of pterocarpanquinones was designed for the new chemical entity (NCE) based on the molecular hybridization of two pharmacophores, quinone, and pterocarpan (derivatives of isoflavonoids) (Fig. 1). Natural quinones represent one of the major classes of natural products with significant biological activity against parasites of the genera *Leishmania*, *Trypanosoma*, and *Plasmodium*. Pterocarpan derivatives, such as maackiain and others, have frequently shown antiprotozoal activity [1]. Two distinct pharmacophoric sites were combined in order to amplify the action spectrum of these groups. This series of derivatives were synthesized through a palladium-catalyzed oxyarylation reaction and screened for their biological activities. Different studies of the group showed a relevant antiparasitic and anti-cancer activity [2–4]. The hit molecule selected LQB-118 (**1**) (Fig. 1) showed antineoplasic activity against cultured breast cancer, leukemia, lung cancer cell lines and prostate cancer cell [5–10], some of which present a Multidrug Resistance phenotype [11]. This hit showed low toxicity for PBMC human blood cells and cell line macrophages, evidencing a high selectivity index [3]. We have also demonstrated that LQB-118 is effective in treating experimental visceral (*Leishmania infantum*) and cutaneous (*L*. *amazonensis* and *L. braziliensis*) leishmaniasis via oral delivery, and therapeutic safety in a repeated toxicity study [12–14]. The death of *L. amazonensis* parasites involved oxidative stress with the hallmarks of apoptosis, similar to cancer-induced death [15]. Despite the development of new therapies for leishmaniasis, in 2015, among the 200 countries or territories reporting to World Health Organization, 87 were identified as endemic for TegumentaryLeishmaniasis (CL) while 75 were considered endemic for Visceral Leishmaniasis (VL) [16]. The clinical forms of the disease, i.e., cutaneous, diffuse, disseminated, mucocutaneous and visceral, are a result of the conjunction of parasite species and the immunological response of patients [17]. It is considered the third most common parasitic disease after schistosomiasis and malaria, based on morbidity and disability-adjusted life years (DALYs) [18]. Besides the fact that the medications (pentavalent antimonials, pentamidine, amphotericin B, liposomal amphotericin B, miltefosine, and paromomycin) are not approved in all countries, leishmaniasis faces the challenge of old and new toxicity concerns with current therapeutic regimens and parasite resistance. If we consider the number of pathogenic species in relation to therapeutic options, this arsenal is still small. Thus, the identification of antileishmanial drug candidates is essential to fill the drug discovery pipeline for leishmaniasis [19]. The biological potential of LQB-118 prompted us to synthesize the second generation of pterocarpanoquinones, which was based on the exchange of position between rings B (pyran) and C (furan), yielding six derivatives 2a-f as shown in Fig. 1, to further investigate the structural features required for the antileishmanial activity. In this paper we report the synthesis of these compounds, the antileishmanial activity on promastigotes and intracellular amastigotes of three species of *Leishmania* (*L. amazonensis*, *L. braziliensis* and *L. infantum*); the selective index was evaluated in murine macrophages and the potential for inducing oxidative stress and alterations in the mitochondrial membrane potential (∆ψm) in the parasite.

## Methods

### Chemistry

Pterocarpanquinones 2a-f were prepared through a palladium-catalyzed oxyarylation of dihydronaphtalen (3a, e, and f) and chromens 3b-d with 3-iodolawsone (4), easily prepared by iodination of lawsone [4, 20–22].

Melting points were determined by a Thomas-Hoover apparatus. Column chromatography was performed on silica gel 230–400 mesh (Aldrich). The ^1^H NMR spectrum was recorded on a Varian (400 or 500 MHz) spectrometer at room temperature. All J values are given in Hz. Chemical shifts are expressed as parts per million downfield shift from tetramethylsilane as an internal standard, and reported as position (δH) (relative integral, multiplicity (s = singlet, d = doublet, dd = double doublet, dt = double triplet, m = multiplet), coupling constant (J Hz) and assignment. The 13C NMR spectrum was recorded on a Varian (100 MHz) spectrometer at room temperature with complete proton decoupling. Data are expressed as parts per million ownfield shift from tetramethylsilane as an internal standard and reported as position (δC).

General procedure for the oxyarylation: Synthesis of compounds type 2. To a stirred solution of 3 (0.33 mmol) and 4 (0.66 mmol) or 5 (0.66 mmol) in acetone (2 mL) or pinacolone (2 mL), silver carbonate (0.5 mmol) and Pd(OAc)_2_ (10 mol%) were added. The reaction mixture was refluxed for 18 h or irradiated for 40 min and filtered in celite with ethyl acetate. The organic layer was washed with brine, dried over anhydrous Na_2_SO_4_ and concentrated. The crude product was washed in n-hexane and purified by flash chromatography on silica.

General procedure for oxyarilations in PEG-400 for syntheses of 2a-f compounds: To 90 mg (0.3 mmol) of 3, 4.6 mg (0.02 mmol) of Pd(OAc)_2_ and 60.7 mg (0.22 mmol) of Ag_2_CO_3_ in 0.4 mL of PEG-400, 0.2 mmol of dihydronaphtalene or chromene was added. After 10 min at 140 °C, TLC analysis showed that all starting materials were consumed. Then, the mixture was filtered in filter paper and transferred to a separation funnel with ethylacetate (20 mL) and washed with brine. (2X 40 mL). After evaporation in the vacuum, the evaporated residue was percolated in silica flash pad using 20% ethyl acetate in hexane as eluent giving the adducts in yields described in Tables 1 and 3.

**Table 1:** Yields and major conditions for reactions shown in Scheme 1

Entry	Heat	Cond. ^a^	2 (%)
1	∆	A	2a (20%)
2	MW ^b^	A	2a (31%)
3	∆	B	2a (57%)
4	MW ^b^	B	2a (50%)
5	∆	C	2a (65%)
6	∆	D	2e (47%)
7	∆	D	2e (46%)

^a^ Conditions: A – Pd(OAc)_2_ (10 mol%), Ag_2_CO_3_ (1.5 equiv.), acetone, reflux, 18 h; B - Pd(OAc)_2_ (10 mol%), Ag_2_CO_3_ (1.5 equiv.), pinacolone, reflux, 18 h; C - Pd(OAc)_2_ (10 mol%), Ag_2_CO_3_ (1.1 equiv.), PEG-400, 140 °C, 10 min.; D - Pd(OAc)_2_ (10 mol%), Ag_2_CO_3_ (1.1 equiv.), PEG-400, 90 °C, 10 min

^b^ MW: 40 W, 60 °C (acetone) or 110 °C (pinacolone), 40 min

### Compound 2a

After column chromatography using n-hexane/ethyl acetate (98:2) as eluant, this compound was obtained as a yellow solid in 57% yield in pinacolone under reflux, mp / at 200 °C. 1H NMR (CDCl3) δ (ppm) 8.09 (ddd, J = 9.2 Hz, 7.5 Hz, 1.4 Hz, 2H); 7.73 (td, J = 7.5 Hz, 1.4 Hz, 1H); 7.68 (td, J = 7.5 Hz, 1.4 Hz, 1H); 7.61–7.55 (m, 1H); 7.33–7.28 (m, 2H); 7.20–7.16 (m, 1H); 5.92 (d, J = 9.8 Hz, 1H); 3.93 (ddd, J = 9.8 Hz, 7.1 Hz, 6.0 Hz, 1H); 2.77 (ddd, J = 15.6 Hz, 8.4 Hz, 3.8 Hz, 1H); 2.66 (ddd, J = 15.6 Hz, 8.4 Hz, 3.8 Hz, 1H); 2.24–2.13 (m, 1H); 2.08–1.96 (m, 1H); 13C NMR (CDCl3, 100 MHz) δ (ppm) 182.4 (C); 177.9 (C); 160.2 (C); 139.3 (C); 134.1 (CH); 133.2 (CH); 132.9 (C); 131.5 (C); 130.9 (CH); 130.5 (CH); 129.0 (CH); 128.4 (CH); 126.9 (C); 126.6 (CH); 126.3 (C); 125.9 (CH); 84.9 (CH); 39.9 (CH); 27.2 (CH2); 25.5 (CH2); MS: m/z 302 (100%), m/z 130 (74%).

### Compound 2b

After column chromatography using n-hexane/ethyl acetate (98:2) as eluant, this compound was obtained as a yellow solid in 30% yield in pinacolone under microwave irradiation, mp / at 190 °C. 1H NMR (CDCl3, 400 MHz) δ (ppm) 8.12–8.06 (m, 2H); 7.75 (td, J = 7.5 Hz, 1.3 Hz, 1H); 7.69 (td, J = 7.5 Hz, 1.3 Hz, 1H); 7.56 (dd, J = 7.6 Hz, 1.3 Hz, 1H); 7.34–7.27 (m, 1H); 7.07 (t, J = 7.6 Hz, 1H); 6.95 (d, J = 8.2 Hz, 1H); 5.90 (d, J = 9.3 Hz, 1H); 4.43 (dd, J = 11.2 Hz, 4.8 Hz, 1H); 4.18 (dd, J = 11.2 Hz, 7.6 Hz, 1H); 4.02 (ddd, J = 9.3 Hz, 7.6 Hz, 4.8 Hz, 1H); 13C NMR (CDCl3, 100 MHz) δ (ppm) 182.1 (C); 177.6 (C); 160.8 (C); 156.2 (C); 134.3 (CH); 133.1 (CH); 132.9 (C); 131.5 (C); 131.1 (CH); 130.8 (CH); 126.4 (CH); 126.0 (C); 123.4 (CH); 122.3 (CH); 118.8 (C); 117.8 (CH); 80.7 (CH); 65.3 (CH2); 40.2 (CH); MS: m/z 304 (26%), 131 (100%).

### Compound 2c

After column chromatography using n-hexane/ethyl acetate (98:2) as eluant, this compound was obtained as a yellow solid in 25% yield in pinacolone under reflux, mp / at 220 °C. 1H NMR (400 MHz, acetone) δ 8.09–8.01 (m, J = 14.0, 7.6 Hz, 2H), 7.90–7.78 (m, 2H), 7.60 (d, J = 2.6 Hz, 1H), 7.34 (dd, J = 8.8, 2.6 Hz, 1H), 6.97 (d, J = 8.8 Hz, 1H), 6.05 (d, J = 9.5 Hz, 1H), 4.41 (dd, J = 11.4, 4.7 Hz, 1H), 4.31 (dd, J = 11.4, 6.9 Hz, 1H), 4.16 (ddd, J = 9.5, 6.9, 4.7 Hz, 1H); 13C NMR (101 MHz, acetone) δ 181.7 (C), 177.1 (C), 160.5 (C), 155.2 (C), 134.2 (CH), 133.2 (CH), 133.0 (C), 131.7 (C), 130.5 (CH), 126.1 (C), 125.8 (CH), 125.5 (CH), 123.2 (C), 121.7 (CH), 119.4 (C), 119.3 (CH), 79.6 (CH), 65.2 (CH2), 40.0 (CH); MS: m/z 340 (15%), 338 (45%), m/z 168 (33%), 166 (100%).

### Compound 2d

After column chromatography using n-hexane/ethyl acetate (98:2) as eluant, this compound was obtained as a light brown solid in 25% yield in acetone under reflux, mp / at 162 °C. 1H NMR (400 MHz, cdcl3) δ 8,09 (dd, J = 5,7 Hz, 2,3 Hz, 2H), 7,78–7,66 (m, 2H); 6,94 (s, 1H); 6,46 (s, 1H); 5,95 (d, J = 0,9 Hz, 1H); 5,93 (d, J = 0,9 Hz, 1H), 5,82 (d, J = 9,3 Hz, 1H); 4,36 (dd, J = 10.9 Hz, 4,8 Hz, 1H); 4,13 (dd, J = 10,9 Hz, 7,5 Hz, 1H); 3,95 (ddd, J = 9,3 Hz, 7,5 Hz, 4,8 Hz, 1H); 13C NMR (101 MHz, acetone) δ 181.7 (C), 177.2 (C), 160.7 (C), 152.1 (C), 149.3 (C), 142.8 (C), 134.2 (CH), 133.1 (CH), 131.7 (C), 125.8 (CH), 125.5 (CH), 123.3 (C), 111.5 (CH), 108.8 (C), 101.6 (CH2), 98.7 (C), 81.1 (CH), 65.3 (CH2), 54.0 (CH), 39.8 (CH).

### Compound 2e

After column chromatography using n-hexane/ethyl acetate (95:5) as eluant, this compound was obtained as a yellow solid in 47% yield in PEG-400 under heating at 90 °C. 1H NMR (400 MHz, CDCl3): δ = 8.08 (t, J = 7.7 Hz, 2 H), 7.76–7.71 (m, 1 H), 7.70–7.64 (m, 1 H), 7.48 (d, J = 8.5 Hz, 1 H), 6.84 (dd, J = 8.4, 2.5 Hz, 1 H), 6.70 (d, J = 2.2 Hz, 1 H), 5.91 (d, J = 9.7 Hz, 1 H), 3.91 (dt, J = 9.7, 6.3 Hz, 1 H), 3.81 (s, 3 H), 2.80–2.68 (m, 1 H), 2.63 (ddd, J = 11.9, 7.0, 4.3 Hz, 1 H), 2.19–2.02 (m, 2 H). 13C NMR (101 MHz, CDCl3): δ = 182.42, 178.03, 160.30, 159.98, 141.20, 134.14, 133.18, 132.92, 132.05, 131.45, 130.23, 126.29, 125.93, 123.21, 113.39, 112.64, 85.27, 55.29, 39.72, 27.46, 25.49. HRMS: m/z [M + Na] + calcd for C21H16O4Na: 355.0940; found: 355.0944.

### Compound 2f

After column chromatography using n-hexane/ethyl acetate (95:5) as eluant, this compound was obtained as a yellow solid in 46% yield in PEG-400 under heating at 90 °C. 1H NMR (400 MHz, CDCl3): δ = 8.09 (ddd, J = 7.2, 5.7, 1.3 Hz, 2 H), 7.74 (td, J = 7.5, 1.4 Hz, 1 H), 7.68 (td, J = 7.5, 1.4 Hz, 1 H), 7.12 (d, J = 2.7 Hz, 1 H), 7.09 (d, J = 8.4 Hz, 1 H), 6.87 (dd, J = 8.4, 2.7 Hz, 1 H), 5.87 (d, J = 9.7 Hz, 1 H), 3.90 (ddd, J = 9.7, 7.7, 5.7 Hz, 1 H), 3.84 (s, 3 H), 2.71 (ddd, J = 15.5, 8.0, 3.8 Hz, 1 H), 2.65–2.56 (m, 1 H), 2.25–2.14 (m, 1 H), 2.02–1.91 (m, 1 H). 13C NMR (101 MHz, CDCl3): δ = 182.37, 177.99, 160.00, 158.34, 134.17, 133.14, 132.96, 131.73, 131.45, 131.16, 129.48, 126.85, 126.31, 125.97, 115.78, 114.51, 84.96, 55.46, 39.83, 26.49, 25.71. HRMS: m/z [M + Na] + calcd for C21H16O4Na: 355.0940; found: 355.0935.

#### Ethics statement

The studies were performed in accordance with protocols approved by the Ethics Committee for Animal Use of the Oswaldo Cruz Foundation (LW07/2010) under the protocol 044/2009, and approved by the Ethics Committee on Animal Use (CEUA) of the Instituto de Biologia Roberto Alcantara Gomes of the Universidade do Estado do Rio de Janeiro-UERJ.

#### Parasites

*Leishmania amazonensis* (MHOM/BR/77/LTB0016) was maintained as promastigotes at 26 °C in Schneider’s insect medium (Sigma-Aldrich) supplemented with 10% heat-inactivated fetal calf serum (HIFCS), streptomycin at 100 μg/mL and penicillin at 100 U/mL. New cultures of promastigotes were obtained from infected Balb/C mice.

*L. braziliensis* (MCAN/BR/98/R619) was routinely isolated from hamster lesions and maintained as promastigotes in Schneider’s insect medium containing 20% HIFCS and 100 μg/ml gentamicin (Schering-Plough). New cultures of promastigotes were obtained from an infected hamster.

*Leishmania infantum* (MHOM/MA67ITMAP263) parasites were isolated from female BALB/c mice infected and cultured at 26 °C in Schneider’s insect medium supplemented with 20% HIFCS, streptomycin at 100 μg/ml and penicillin at 100 U/ml. New cultures of promastigotes were obtained from infected Balb/C mice.

#### Antipromastigote test

*Leishmania* promastigotes of three species were incubated in 96-well plates (Nunc, Roskilde, Denmark) under the conditions described above in either the absence or presence of different concentrations of pterocarpanquinones 2a-f (0-100 μM). A stock solution of the compounds was prepared at 50 mM in dimethylsulfoxide (Sigma Aldrich). The maximum solvent concentration used in the assays was 0.4% in a final volume per well of 200 μL. The cultures were initiated with 1.0 × 10^6^ cells/ml and maintained at 26 °C for 72 h. Inhibition of parasitic growth was determined by cell viability indicator, resazurin [23], with excitation λ = 560 nm and emission λ = 590 nm or the number of parasites was then counted in a Neubauer chamber (experiments with *L. braziliensis*). The 50% inhibitory concentration (IC_50_) was determined by nonlinear regression analysis in the software GraphPad Prism 5.

#### Antiamastigote test

For the test against intracellular amastigotes of *L. amazonensis* and *L. infantum*, resident peritoneal macrophages (swiss mouse) were plated in RPMI (Sigma-Aldrich) at 2 × 10^6^/mL (0.4 mL/well) in Lab-Tek eight-chamber slides (Nunc, Roskilde, Denmark) and incubated at 37 °C in 5% CO_2_ for 1 h. Stationary-phase *L. amazonensis* and *L. infantum* promastigotes were added at respective parasite/macrophage ratios of 3:1 and 5:1 for 4 h. After washing three times, pterocarpanquinones 2a-f at various concentrations were added for 72 h. Next, the slides were stained using an Instant Prov hematological dye system (Newprov, Curitiba, Brazil). The number of amastigotes was determined by counting at least 100 macrophages per sample.

Macrophages of golden hamsters were used in assays against intracellular amastigotes of *L. braziliensis* due to the natural resistance of mice. Resident macrophages were obtained from the peritoneal cells of golden hamsters after a peritoneal injection of 10 mL of Dulbecco’s Modified Eagle’s medium (DMEM) (Sigma-Aldrich). The peritoneal macrophages were plated onto glass coverslips placed within the wells of a 24-well culture plate and incubated at 37 °C in 5% CO_2_ for 1 h. After removing the nonadherent cells, the monolayers were infected with stationary-phase *L. braziliensis* promastigotes at a 5:1 parasite/macrophage ratio for 4 h. The infected macrophages were washed and incubated with several pterocarpanquinone concentrations (0-25 μM) for 72 h. The monolayers were then stained with Giemsa, and at least 100 infected macrophages per sample were counted under optical microscopy.

The results were expressed as an infection index (% infected cells×number of amastigotes/total number of macrophages). The 50% inhibitory concentration (IC_50_) was determined by nonlinear regression analysis in the software GraphPad Prism 5.

#### Macrophage toxicity test

To evaluate the toxicity of pterocarpanquinones 2a-f, peritoneal macrophages of Swiss mice (2 × 10^6^/mL) were incubated with pterocarpanquinones (1.25–100 μM) for 72 h at 37 °C/5% CO_2_. Viability was evaluated with resazurin assay as described for the antipromastigote test. The 50% cytotoxicity concentration (CC_50_) was determined by nonlinear regression analysis in the software GraphPad Prism 5.

#### Measurement of reactive oxygen species

Intracellular ROS levels were measured in treated and untreated cells as described previously [15]. Briefly, 1 × 10^7^ promastigotes/mL of *L. infantum* were incubated in Schneider’s insect medium supplemented with 20% HIFCS at 26 °C with concentrations of pterocarpanquinones 2a-f ranging from 0 to 5 μM in the presence of 20 μM H_2_DCFDA (Molecular Probes, Eugene, OR, USA). The fluorescence was monitored at 1 h intervals for 4 h using 485 and 530 nm as excitation and emission wavelengths, respectively, using the spectrofluorometer Spectra Max Gemini XPS (Molecular Devices, Silicon Valley, CA, USA). Antimycin A 10 μM was used as positive control.

#### Mitochondrial membrane potential (ΔΨm) test

To determine the effect of pterocarpanquinones 2a-f on the ΔΨm, promastigotes of *L. infantum* (5 × 10^6^ cells/mL) were incubated in the presence of 0–5 μM of derivatives at 26 °C. After 4 h, the parasites were incubated for 15 min with 10 μg/mL rhodamine 123 (Rh123) (Sigma-Aldrich). Data acquisition of 10,000 events and analysis were performed using the flow cytometer FACSCalibur (Becton-Dickinson, Rutherford, NJ, USA). Alteration in ΔΨm was quantified using an index of variation (IV) obtained by the eq. IV = (MT − MC)/MC [24], where MT is the median of the fluorescence of Rh123 for treated parasites and MC is the median of the fluorescence of the control parasites. Negative IV values correspond to depolarization of the mitochondrial membrane. The FCCP (Carbonyl cyanide-*p*-Trifluoromethoxyphenylhydrazone) 20 μM was used as positive control [25].

#### Statistical analysis

Antipromastigote and macrophage toxicity assays were repeated three times in triplicate. Antiamastigote assays were repeated three times in duplicate. The results reported for these assays are presented by the Mean ± SEM. Measurements of ROS and ΔΨm were performed in triplicate three times. The ROS and index of variation data of the Ψm are represented as Mean ± SD. Significant differences between pairs of groups were assessed using Student’s t-test with the significance level set at *p* < 0.05. Dose-response significance was evaluated by one-way ANOVA test.

## Results

### Chemistry

**Scheme 1:**
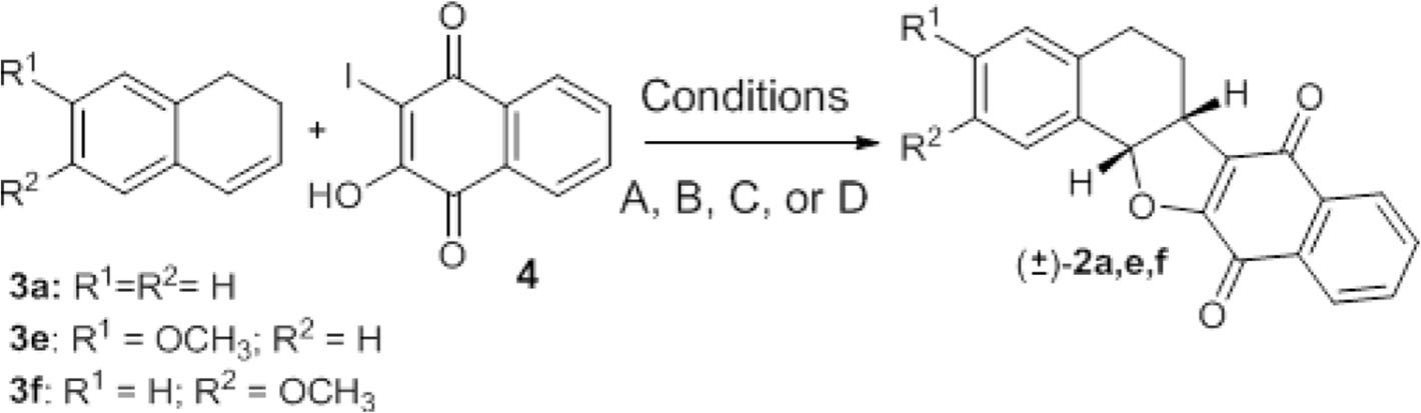
Palladium-catalyzed oxyarylation of 3a, e, and f with 4

Pterocarpanquinones 2a-f were prepared through a palladium-catalyzed oxyarylation of dihydronaphtalen (3a,e, and f) and chromens 3b-d with 3-iodolawsone (4), easily prepared by iodination of lawsone under three reaction conditions (A, B, C, and D). Herein, we used the oxyarylation to obtain type 2 compounds. We first studied the reaction between 3a and 4 that leads to pterocarpanquinone 2a (Scheme 1, Table 1). Using acetone as solvent under thermal conditions (Condition A, Table 1, entry 1) 2a was obtained in 20% after 18 h of reaction. The reaction was faster under microwave heating (40 min.), producing 2a at a 31% yield (Table 1, entry 2). Following the classic experimental conditions for a cationic pathway in palladium-catalyzed oxyarylation [26, 27], when 3 equiv. of Ag_2_CO_3_ was used, did not improve 2a yields. Therefore, we decided to run the others experiments using 1.5 times the previous salt amount. The yield additionally increased when pinacolone (bp 110 °C) was used as the solvent instead of acetone. Under conventional heating, a 57% yield was obtained from 2a after 18 h of reaction (entry 5) while under microwave irradiation 2a was isolated at 50% yield after 40 min of reaction. When PEG-400 was utilized as solvent at 140 °C, 3a reacts with 4 and 2a could be isolated at a 65% yield after 10 min. of reaction (Table 1, entry 5), which meant a yield increase when compared with acetone or pinacolone under reflux, using a microwave or not, in little time.

**Scheme 2:**
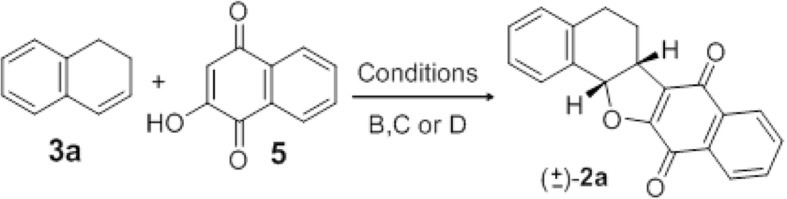
Dehydrogenative Heck reaction experiments of 3a with 5

In fact, pterocarpanquinone 2a could also be obtained by this unprecedented oxyarylation of 3a with lawsone 5 (Scheme 2). Under thermal conditions, 2a was obtained at 34% yield (entry 1, Table 2). When PEG-400 is used, in 40 min much degradation of materials was observed and 2a was formed in traces (entry 2, Table 2).

**Table 2:** Yields of dehydrogenative Heck Reaction experiments

Entry	Cond. ^a^	Time	Oxidant	2a (%)
1	B	18 h		34%
2	C	2.5 h		Traces ^b^
3	D	18 h	Cu(AcO)_2_	30%
4	D	40 min	K_2_S_2_O_8_	15%

^a^ Conditions: B - Pd(OAc)_2_ (10 mol%), Ag_2_CO_3_ (1.5 equiv.), pinacolone, reflux, C - Pd(OAc)_2_ (10 mol%), Ag_2_CO_3_ (1.1 equiv.), PEG-400, 140 °C. Conditions: D - Pd(OAc)_2_ (10 mol%), Ag_2_CO_3_ (1.5 equiv.), Oxidant, pinacolone, reflux

^b^ 2a’ was formed in 60:40 proportion 2a:2a’, respectively

**Scheme 3:**
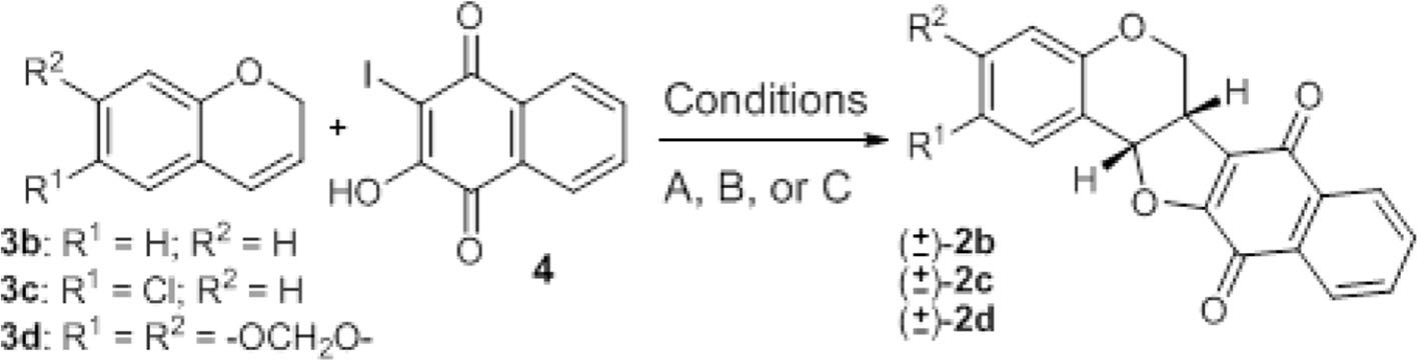
Synthesis of pterocarpanquinones 2b-d

In addition to 2a, we could have isolated and identified by ^1^H NMR and through 2D NMR techniques the regioisomer 2a’, which did not appear in previous experiments (Scheme 3). The pterocarpanquinones 2e and f were obtained at respective yields of 47 and 46% using PEG-400 as solvent at a lower temperature (90 °C, Table 1, entries 6 and 7, Condition D). This type of palladium-catalyzed reaction, also called the dehydrogenative Heck reaction, is in general improved in the presence of an oxidant, to transform Pd[0] generated at the end of the catalytic cycle to Pd[2+]; but unfortunately in our case, the use of Cu(OAc)^2^ and K_2_S_2_O_8_ (entries 3 and 4, Table 2) did not lead to better chemical yields. Once better yields were obtained from the oxyaryation using 4 instead of 5, this compound was used in the next experiments. The oxyarylation of chromens 3b-d with 4 (Scheme 3) led to pterocarpanquinones 2b-d. Two reaction conditions were used, B (acetone) and C (PEG-400). Compound 2b was prepared at 30% using Condition B while in Condition C, the yield slightly decreases to 26% yield (entries 1 and 2). For chromens 3c, the yield increased from 25 to 75% when going from conditions B to C (entries 3 and 4). Finally, Compound 2c was formed by oxyarylation of 4 with 3d, at a moderate yield (40%) (entry 5) under Condition C (Table 3).

**Table 3:** Yields and conditions for 2b-d

Entry	Cond. ^a^	3	2 (%)
1	B ^b^	3b R^1^ = R^2^ = H	2b (30%)
2	C	3b R^1^ = R^2^ = H	2d (26%)
3	B	3c R^1^ = Cl; R^2^ = H	2c (25%)
4	C	3c R^1^ = Cl; R^2^ = H	2c (72%)
5	C	3d R^1^ = R^2^ = -OCH_2_O-	2d (40%)

^a^ Conditions: B - Pd(OAc)_2_ (10 mol%), Ag_2_CO_3_ (1.5 equiv.), pinacolone, reflux, 18 h; C - Pd(OAc)_2_ (10 mol%), Ag_2_CO_3_ (1.1 equiv.), PEG-400, 140 °C, 10 min

^b^ MW: 40 W, 60 °C,110 °C, 40 min

### Antipromastigote activity

*L. amazonensis*, *L. braziliensis*, and *L. infantum* promastigotes were incubated with different concentrations of the pterocarpanquinones for 72 h and the viability was evaluated. The potency of the derivatives in inhibiting the proliferation of *L. amazonensis* and *L. infantum* promastigotes was similar; nevertheless, *L. braziliensis* had less susceptibility to the pterocarpanquinones. Compared with LQB-118, the compounds 2a-f were more potent against *L. amazonensis* and *L. infantum*; however, they presented lower potency against *L. braziliensis* (Table 4).

**Table 4:** The action spectrum of second-generation pterocarpanquinones on *Leishmania* and toxicity in macrophages

Compounds	Murine Macrophage	*L. amazonensis*			*L. braziliensis*			*L. infantum*		
		Promastigote	Amastigote	SI ^d^	Promastigote	Amastigote	SI ^d^	Promastigote	Amastigote	SI ^d^
	CC_50_ (μM)	IC_50_ (μM)			IC_50_ (μM)			IC_50_ (μM)		
LQB 187 (2a)	49.30 ± 1.10	1.05 ± 0.21	0.90 ± 0.10	54.7	23.10 ± 1.63	9.85 ± 1.37	5.0	1.20 ± 0.20	> 25	ND
LQB 182 (2b)	16.90 ± 1.20	1.08 ± 0.20	0.85 ± 0.02	19.8	10.98 ± 1.25	7.84 ± 2.46	2.15	1.00 ± 0.30	3.60 ± 0.90	4.69
LQB 236 (2c)	43.90 ± 0.60	1.15 ± 0.18	0.60 ± 0.13	73.16	17.85 ± 1.12	8.34 ± 1.47	5.26	1.80 ± 0.30	> 25	ND
LQB 168 (2d)	77.70 ± 1.10	1.37 ± 0.04	0.45 ± 0.06	172.6	28.21 ± 1.61	7.04 ± 2.29	11.03	2.00 ± 0.40	> 50	ND
LQB 474 (2e)	14.92 ± 1.43	0.50 ± 0.07	2.60 ± 0.8	5.74	12.34 ± 0.45	8.53 ± 1.12	1.74	2.20 ± 0.30	> 10	ND
LQB 475 (2f)	49.90 ± .1.00	0.40 ± 0.06	2.10 ± 0.4	23.76	10.50 ± 1.01	9.02 ± 1.64	5.53	1.40 ± 0.70	> 40	ND
LQB-118 (1)	18.46 ^a^	1.73 ^a^	1.45 ^a^	12.73	3.40 ^b^	7.50 ^b^	2.46	4.08 ^c^	3.25 ^c^	5.68
Pentamidine	8.50 ± 1.25	4.80 ± 0.09	1.90 ± 0.10	4.47	13.0 ± 0.04	7.70 ± 2.40	1.1	5.70 ± 0.12	0.40 ± 0.20	21.25

*ND* Not Determined

^a^ reference [14]

^b^ reference [13] e

^c^ reference [12]

^d^ Selective Index (SI) = CC_50_ in Macrophages/IC_50_ in intracellular amastigotes

### Antiamastigote activity and selectivity

To evaluate the activity of derivatives against intracellular amastigotes, which correspond to the clinically relevant form of the parasite, peritoneal macrophages were infected with three species of *Leishmania* and incubated for 72 h with different concentrations of the pterocarpanquinones. The compound **2d** (methylenedioxy group in ring D) showed the lowest IC_50_ (0.45 μM for *L. amazonensis*; 7.04 μM for *L. braziliensis*) for the species causing tegumentary leishmaniasis, which is in relation to second generation derivatives and LQB-118. In addition to improved potency, derivative **2d** showed less toxicity to peritoneal macrophages, with a 4.2-fold lower cytotoxic concentration than LQB-118 (CC_50_ = 77.7 μM and CC_50_ = 18.46 μM). This decrease in cytotoxicity aligned with increased potency of pterocarpquinone **2d** resulted in a selectivity index of 172.6 for *L. amazonensis*. Despite the potency in intracellular amastigotes of *L. amazonensis*, compound **2d** showed no significant activity in *L. infantum* amastigote. Only derivative **2b** displayed activity against the three species despite the low selectivity index for *L. braziliensis* and *L. infantum*, as presented by the reference drug pentamidine, which showed low index selectivity for L. amazonensis and *L. braziliensis*, and 10-fold greater selectivity (Table 4).

### Pterocarpanquinones induce oxidative stress in *L. infantum* promastigotes

**Fig. 2:**
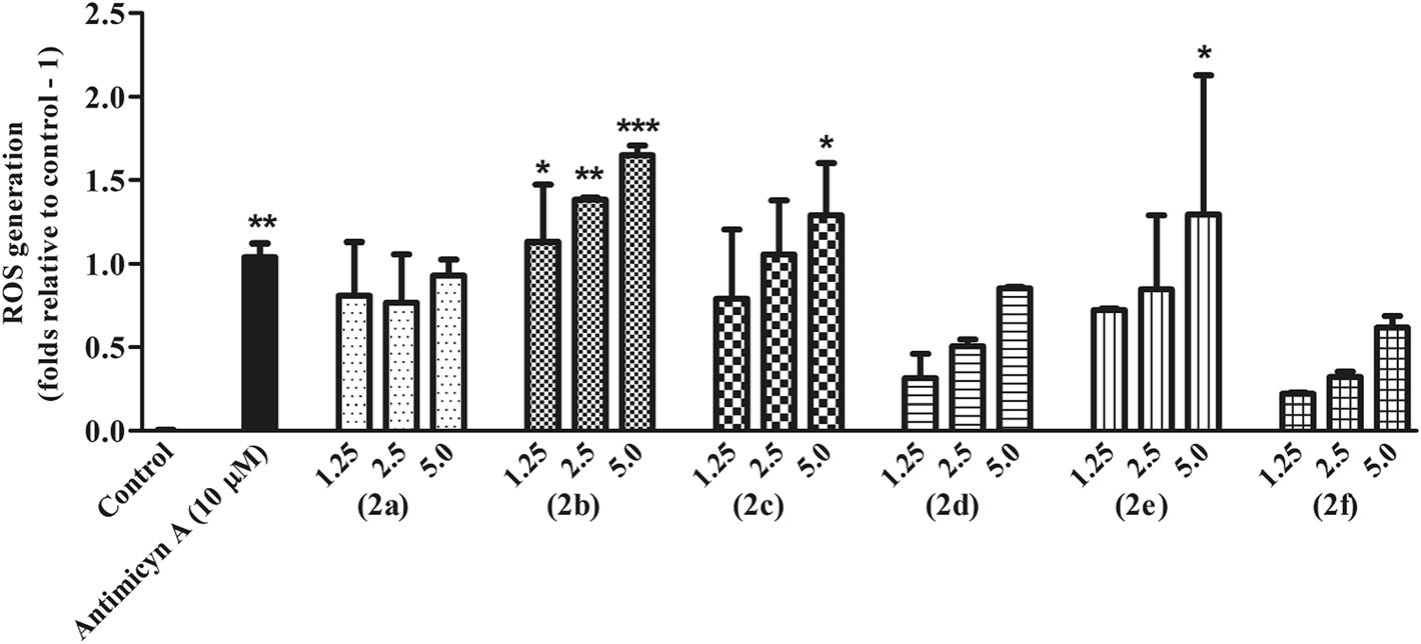
Early ROS production in L. infantum promastigotes treated with pterocarpanquinones. Promastigotes at 1x107cells/mL were incubated with 1.25 and 2.5 μM of pterocarpanquinones; ROS production was monitored for 4 h with 20 μM of the H2FDCDA, with excitation at 485 nm and emission at 530 nm. The graph represents the moment 4 h after incubation. *p < 0.05, ** *p* < 0.01 and ***p < 0.001 compared with control

Early Reactive Species Oxygen (ROS) production in promastigotes of *L. infantum* treated with second-generation pterocarpanquinones (1.25–5.0 μM) was evaluated with the probe H_2_DCFDA for 4 h. The production of ROS after 4 h of treatment was significant for compounds **2b**, **2c**, and **2e** (Fig. 2). The pterocarpanquinone **2b** increased ROS generation dependent on compound concentration, whereas compounds **2c** and **2e** only increased ROS production when the parasite was incubated with the highest concentration of 5 μM. These results indicate that the presence of pyran and the absence of clusters in ring D in R_1_ or R_2_ may influence the induction of ROS production.

### *L. infantum* promastigotes altered mitochondrial membrane potential after treatment with pterocarpanquinones

**Fig. 3:**
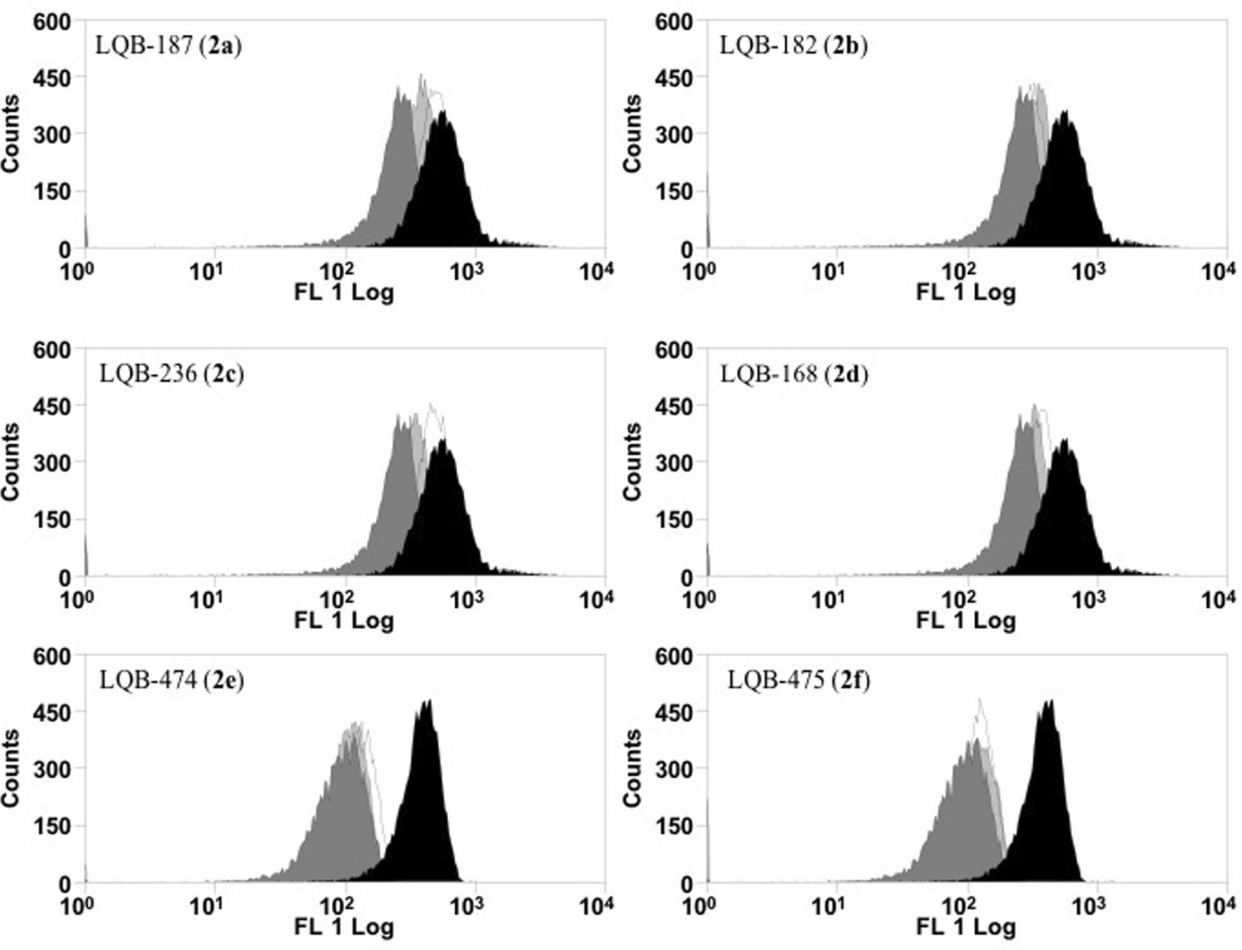
L. infantum promastigotes altered mitochondrial membrane potential after treatment with pterocarpanquinones. Promastigotes of L. infantum (5 × 106 cells/mL) were cultured in the presence of 0–2.5 μM of derivatives at 26 °C. After 4 h, the parasites were incubated for 15 min with 10 μg/mL rhodamine 123 (Rh123). Data acquisition and analysis were performed using a FACSCalibur flow cytometer. The FCCP 20 μM was used as positive control. Black (control); White (1.25 μM); Light Gray (2.5 μM) and Dark Gray (FCCP)

Promastigotes treated with second-generation pterocarpanquinones for 4 h had their mitochondrial membrane potential (ΔΨm) evaluated with rhodamine 123 by flow cytometry. The displacement of populations of treated cells, to the left in the histogram, represents the depolarization of the cells, as verified with the positive control used (FCCP), a classical uncoupler of ΔΨm (Fig. 3). All pterocarpquinones altered the ΔΨm; however, by analysis of the variation index in Table 5, compounds **2e** and **2f** induced greater depolarization in *L. infantum* promastigotes.

**Table 5:** Analysis of Δѱm. index of variation (IV) of *L. infantum* promastigotes treated with LQBs

Compounds	μM	Index of variation ^a^
Control	0	0
LQB 187 (2A)	1.25	−0.103 ± 0.16
	2.5	−0.368 ± 0.03
LQB 182 (2B)	1.25	−0.397 ± 0.07
	2.5	−0.402 ± 0.04
LQB 236 (2C)	1.25	−0.360 ± 0.16
	2.5	−0.258 ± 0.17
LQB 168 (2D)	1.25	−0.369 ± 0.08
	2.5	−0.194 ± 0.18
LQB 474 (2E)	1.25	−0.582 ± 0.1
	2.5	−0.701 ± 0.01
LQB 475 (2F)	1.25	−0.668 ± 0.01
	2.5	−0.707 ± 0.01
FCCP	20	−0.727 ± 0.01

Values refer to the mean and standard deviation of three experiments

*MT* median fluorescence of treated parasites, *MC* median fluorescence of control parasites

^a^ The changes in the fluorescence intensity of rhodamine 123 were quantified from the index of variation obtained by the equation (MT – MC) / MC

## Discussion

Limited therapeutic options in leishmaniasis make its treatment very challenging. This small arsenal has resulted in long-term treatment and severe adverse effects. It is still important to emphasize that there are no vaccines or chemoprophylaxis currently available for humans [28, 29]. Given the therapeutic scenario for leishmaniasis, which includes a spectrum of diseases caused by more than 20 *Leishmania* species found in many regions of the world [30], there is a need for new drugs that provide efficacy, safety, low cost, oral bioavailability, and action against resistant strains. In this context, the first-generation pterocarpanquinones were designed based on the molecular hybridization of two pharmacophores, quinone, and pterocarpan (derivatives of isoflavonoids). This series was synthesized through a new palladium-catalyzed oxyarylation (oxa-Heck) reaction of chromenquinone with ortho-iodophenol in a four-step production process [2]. The synthesis of the first- and second-generation pterocarpanquinones is in accordance with the proposal of Katsuno *et. al*., which establishes some generic hit selection criteria for infectious diseases, namely that the hit compound should ideally be synthesized in up to five steps with an acceptable yield and acceptable solubility [31]. Recent studies have shown that LQB-118 (the first generation of pterocarpanquinones) is a suitable structure for the development of promising drugs for the oral treatment of leishmaniasis [12]; thus, the second generation was produced and its activity spectrum was tested on two species that cause tegumentary leishmaniasis (*L. amazonensis* and *L. braziliensis*) and a species that causes visceral leishmaniasis (*L. infantum*). In this work, we answered two pertinent questions about the structure of the first generation pterocarpanquinones: (i) the configuration of the position of rings B (pyran) and C (furan) of the pterocarpan core and (ii) the presence of oxygen in the B ring. When rings B and C are inverted (**2b** in comparison to **1**), we noted an improvement of about 1.6 times in the activity against promastigotes and amastigotes of *L. amazonensis* and 4 times against promastigotes of *L. infantum*. Notwithstanding, the opposite was observed in promastigotes of *L. braziliensis*, which presented a decrease of 3.2 times in the antileishmanial activity with the second generation compound. As to the oxygen in the ring B of the new generation, we observed that the oxygenated compound **2b** is approximately two times more active against promastigotes of *L. braziliensis* than its deoxy derivative **2a**. Furthermore, on average, the oxygenated compounds **2b**, **2c** and **2d** were more potent against amastigotes of *L. amazonensis* than the deoxy derivatives **2a**, **2e**, and **2 f**. Another modification that improved the activity was the addition of the methylenedioxy group (**2d**). This group has demonstrated the ability to potentiate the activity of triazole compounds [32], analogs of camptothecin [33], thiosemicarbazones [34] and secondary metabolites isolated from the stem bark of *Rollinia emarginata* [35]. We verified this potential in the phenotypic trials on *L. amazonensis* and *L. braziliensis*; however, this effect was not seen in promastigotes of *L.infantum*. The chlorine atom as the substituent in R2 in **2c** increased cytotoxicity when compared to **2b** by 2.6 times; this increased cytotoxicity profile was also observed with the insertion of one or two chlorine atoms in the 4-Phenyl-1,3-thiazol-4-amines series [36]. Croft and cols reported that *Leishmania* species show a significant variation in their sensitivity to established and experimental drugs [37]. As expected, variation in the susceptibility among species was evident in the clinically relevant form of the parasite, the intracellular amastigote. *L. amazonensis* was the species most susceptible to novel derivatives, whilst *L. infantum* was resistant to most of them. In fact, the compound 2b was the only one active against *L. infantum* amastigotes. The mechanism of induction by pterocarpanquinone LQB-118 of *L. amazonensis* parasite death involved oxidative stress with hallmarks of apoptosis, similar to cancer-induced death [15]. The second-generation pterocarpanoquinones (**2b** and **2c**) that possess the oxygen atom in ring B, showed induction of increased ROS production. However, the pterocarpanquinone (**2d**) with methylenedioxy substituent altered this ROS-increasing ability, as also presented by the pterocarpanquinones with an absence of oxygen in the B (**2a**, **2e**, and **2f**). Natural and synthetic quinones may undergo redox cycling and induce oxidative stress with ROS production [24, 38].

## Conclusion

The data presented indicate that the second-generation pterocarpanoquinones are promising scaffolds for the development of new leishmanicidal agents.
